# Polylysine-based copolymer self-assemblies featuring acidity-activated structural transformation perceives and relieves sepsis

**DOI:** 10.1093/rb/rbaf100

**Published:** 2025-09-29

**Authors:** Qilong Wu, Chao Fang, Taixia Wang, Qiuxia Peng, Kun Zhang, Dan Wang, Shihao Xu

**Affiliations:** Department of Ultrasound Medicine, The First Affiliated Hospital of Wenzhou Medical University, Wenzhou, Zhejiang 325000, China; Department of Orthopedics and Central Laboratory, Sichuan Academy of Medical Sciences, Sichuan Provincial People’s Hospital, School of Medicine, University of Electronic Science and Technology of China, Chengdu, Sichuan 610072, China; Department of Orthopedics and Central Laboratory, Sichuan Academy of Medical Sciences, Sichuan Provincial People’s Hospital, School of Medicine, University of Electronic Science and Technology of China, Chengdu, Sichuan 610072, China; Department of Comprehensive Oncology Center, Shanghai Pulmonary Hospital, Tongji University Medical School Cancer Institute, School of Medicine, Tongji University, Shanghai 200433, China; Department of Medical Ultrasound, Shanghai Tenth People’s Hospital, School of Medicine, Tongji University, Shanghai 200072, China; Department of Orthopedics and Central Laboratory, Sichuan Academy of Medical Sciences, Sichuan Provincial People’s Hospital, School of Medicine, University of Electronic Science and Technology of China, Chengdu, Sichuan 610072, China; Department of Orthopedics and Central Laboratory, Sichuan Academy of Medical Sciences, Sichuan Provincial People’s Hospital, School of Medicine, University of Electronic Science and Technology of China, Chengdu, Sichuan 610072, China; Department of Ultrasound, Shanghai Municipal Hospital of Traditional Chinese Medicine, Shanghai University of Traditional Chinese Medicine, Shanghai 200071, China; Department of Ultrasound Medicine, The First Affiliated Hospital of Wenzhou Medical University, Wenzhou, Zhejiang 325000, China; National Key Clinical Specialty (Wound Healing), The First Affiliated Hospital of Wenzhou Medical University, Wenzhou, Zhejiang 325000, China; Wenzhou Key Laboratory of Interventional Ultrasound for Intelligent Healthcare and Clinical Translation, The First Affiliated Hospital of Wenzhou Medical University, Wenzhou, Zhejiang 325000, China

**Keywords:** sepsis, self-assembly, acidity-activated structural transformation, sepsis pathogenesis targeting, sepsis monitoring

## Abstract

Sepsis, a systemic inflammatory response syndrome, causes severe immune dysfunction and is associated with high mortality because of the lack of effective clinical interventions. To address the pathogenesis of sepsis, such as bacterial infection and the exacerbation of inflammation and oxidative stress, an acidity-activated polylysine (PLL)-based copolymer self-assembly (PPDD) was developed. This material was synthesized by conjugating polyethylene glycol-modified PLL (PEG-PLL) with 2,7-dichlorofluorescein diacetate (DCFH-DA). PPDD, with its PLL-derived antibacterial and antioxidant properties, can scavenge reactive oxygen species (ROS), mitigate inflammation and eliminate bacteria. These combined actions help alleviate the symptoms of sepsis and improve survival rates. *In vitro* and *in vivo* experiments confirmed that this approach can rapidly neutralize ROS, significantly reduce pro-inflammatory cytokine cascades and effectively clear bacteria, thereby improving physiological stability and survival rates. Notably, Day-14 survival reached 80% in the PPDD-treated group compared with 20% in septic controls. More significantly, when the PPDD copolymer self-assembles into the acidic sepsis microenvironment, it disassembles and reconfigures from a spherical to an ellipsoidal structure. This acidity-activated structural transformation exposes more bioactive components for ROS scavenging, which is beneficial for removing oxidative stress, killing bacteria, reducing inflammation and alleviating sepsis. Following PPDD administration, systemic levels of TNF-α, IL-6, IL-10 and CRP were reduced by 38.1%, 46.0%, 76.7% and 32.9%, respectively, confirming its robust anti-inflammatory effect. Additionally, the conjugated DCFH-DA, a cell-permeable fluorescent probe, enables monitoring of oxidative stress and tracing the evolution of sepsis, especially after treatment. A comprehensive biosafety assay revealed no detectable hemolysis or organ toxicity, substantiating the translational potential of this platform. Our biocompatible and acidic sepsis environment-responsive PPDD paves a solid foundation for the clinical diagnosis and treatment of sepsis.

## Graphical abstract

**Figure:**
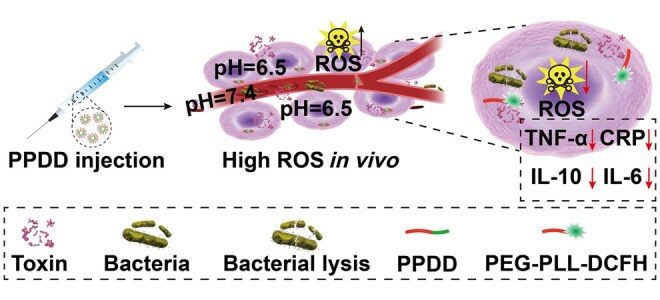

## Introduction

Sepsis is a life-threatening systemic inflammatory response syndrome that poses a serious threat to human health. Due to the lack of effective clinical interventions, patients often experience immune dysfunction, which leads to excessive systemic inflammation and multi-organ dysfunction. While current clinical interventions such as fluid resuscitation, mechanical ventilation, anti-infection therapy and hemodynamic support can partially ameliorate symptoms, the mortality rate among patients with sepsis remains unacceptably high. Sepsis is often characterized by elevated levels of reactive oxygen species (ROS), including hydroxyl radicals and superoxide anion, leading to significant damage to the body [1–3]. These ROS can cause lipid peroxidation of cell membranes [4–6], DNA breakage [7–9] and protein structural alterations [10], resulting in disturbances of the intracellular milieu and subsequent cellular dysfunction. Additionally, these ROS have the ability to induce severe inflammation and impair the normal immune function, rendering the body more vulnerable to subsequent microbial attacks [11, 12]. Therefore, it is crucial not only to control the source of infection but also to actively eliminate endogenous ROS, which is expected to promptly restore damaged tissues and normal physiological function when treating sepsis [13, 14]. Antioxidant therapy is an advanced clinical approach aimed at reducing the harmful effects of free radicals on cellular and tissue integrity by enhancing the body’s antioxidant capacity [15–17], showing significant potential in alleviating inflammation- and ROS-related diseases, such as sepsis [18]. By scavenging oxidative stress, antioxidant therapy has the potential to improve the patients’ physiological well-being and is gaining increasing attention, especially in clinical practice [19–23]. Therefore, antioxidant therapy is expected to benefit a wide range of patients with sepsis [18, 24]. However, traditional antioxidants have been found to exhibit low structural stability, limited scavenging activity toward diverse ROS and reactive nitrogen species (RNS) and short-lived activity under physiological conditions [25]. More significantly, they are also unable to monitor sepsis evolution, especially variations in prognosis. Consequently, they are unable to effectively inhibit the dysregulated inflammatory responses in sepsis, let alone monitor disease progression and prognosis.

Currently, a wide range of inorganic nanomaterials have emerged as promising therapeutic agents for treating ROS-associated diseases [26–28], such as nanozymes and single-atom nanozymes (SAZymes) [29–32]. These innovative nanozyme-based nanomaterials provide an alternative approach to addressing ROS-related ailments, opening up new opportunities for medical intervention; nevertheless, they suffer from unresolved biosafety and biotoxicity concerns because the dominant active components of most nanozymes and SAZymes are transition metals with high atomic numbers. Instead of relying solely on inorganic antioxidants, a wide variety of small natural molecules, such as vitamin derivatives and N-acetylcysteine, are utilized as antioxidants in the treatment of sepsis [19, 33]. These small organic compounds reduce free-radical formation, thereby protecting cells from oxidative damage and playing a crucial role in the therapy.

**Figure 1.**
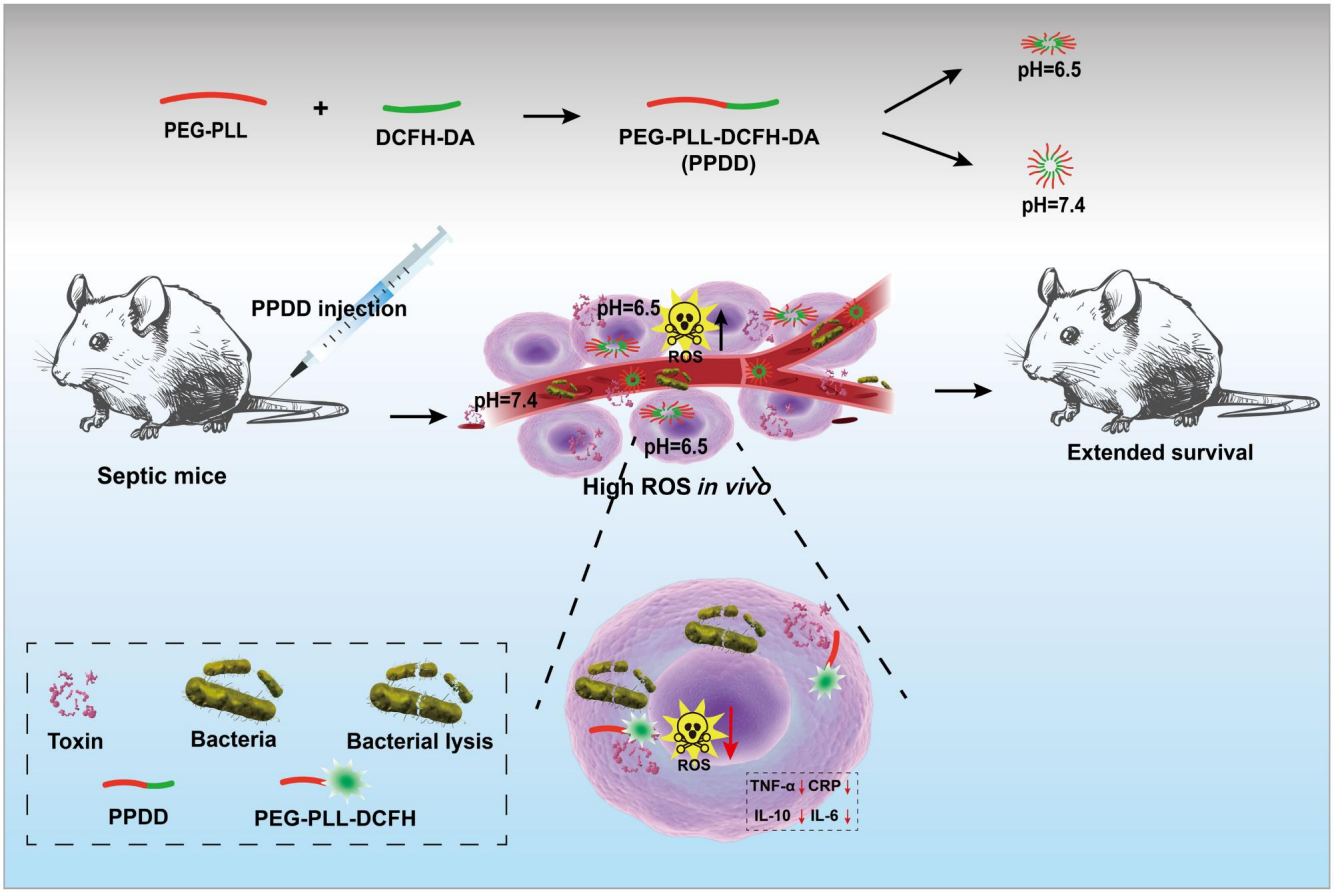
Synthesis and action principles of PPDD for removing oxidative stress, killing bacteria, attenuating inflammation and treating sepsis.

In this study, an acidity-activated polylysine (PLL)-based copolymer self-assembly was obtained by conjugating PEG-modified PLL (PEG-PLL) with 2,7-dichlorofluorescein diacetate (DCFH-DA), and is hereafter referred to as PEG-PLL-DCFH-DA (PPDD) (Figure 1). PLL is an antimicrobial peptide (AMP) produced by Streptomyces [34]. It consists of a polymeric chain of 25–35 lysine residues. As a naturally occurring antibacterial and antioxidant compound, PLL exhibits broad antibacterial activity against a variety of microorganisms, including Gram-positive and Gram-negative bacteria, yeast and fungi [35]. Moreover, the bactericidal properties of ROS-scavenging PLL effectively accelerated recovery from bacterial infections by eradicating bacteria and suppressing inflammation [36–41]. Inspired by them, PPDD is expected to scavenge ROS, mitigate inflammation and kill infected bacteria, which will undoubtedly benefit sepsis alleviation and improve the survival rate. More significantly, under neutral conditions, spherical self-assemblies were formed, whereas, once they entered acidic milieu, structural transformation or collapse occurred. Such a structural transformation process favored greater bioactive components exposure, benefiting ROS scavenging, bacterial killing, inflammation mitigation and sepsis alleviation, given the acidic microenvironment characteristic of sepsis. Recent polymeric nanoparticle systems for sepsis include hydrogel nanogels that neutralize extracellular histones [42], telodendrimer-derived “nanotraps” that adsorb cytokines and endotoxins [43], and polydopamine nanoparticles that scavenge reactive oxygen species and cell-free DNA [44]. These platforms still present biosafety concerns or provide limited protection. In contrast, PPDD shows high biocompatibility and uses acidity induced exposure of antioxidant groups to enhance reactive oxygen species clearance, leading to markedly improved survival in septic models. Additionally, DCFH-DA is a cell-permeable fluorescent probe for detecting ROS [21], which, therefore, enables oxidative-stress monitoring to track sepsis progression and evaluate treatment efficacy. Collectively, this research protocol not only reduces the inherent biotoxicity of PLL but also effectively decreases intracellular ROS levels and achieves seamless integration of sepsis diagnosis and therapy with prognosis monitoring. The PPDD developed by our team shows great promise in providing a novel platform to clinical diagnosis and treatment of sepsis.

## Materials and methods

### Materials

Human umbilical vein endothelial cells (HUVEC) and macrophages were obtained from the cell bank of Central Laboratory of Sichuan Academy of Medical Sciences (Chengdu, China). DMEM high-glucose medium, penicillin and phosphate-buffered saline (PBS) were purchased from Solarbio (China). In addition, 100% of fresh fetal bovine serum (FBS) was purchased from Gibco (America). Polylysine PEG Conjugate (PEG-PLL) and ethanol were acquired from Shanghai Aladdin Biochemical Technology Co., LTD. DCFH-DA and other reagents were sourced from Shanghai Biyuntian Biotechnology Co., LTD. Female athymic BALB/c mice were provided by Beijing Weitong Lihua Laboratory Animal Technology Co (Beijing, China).

### Methods

#### Synthesis of PPDD

A reaction bottle was charged with 0.25 g of DCFH-DA, 0.2 g of 3-hydroxy-2-butanone, 0.083 g of 1-hydroxybenzotriazole, and 0.2 g of N,N-diisopropylethylamine. N,N-dimethylformamide was added as the solvent. The mixture was stirred in an ice bath for 30 min. Subsequently, 0.8g of PEG-PLL material was added to the reaction flask and stirred at room temperature for 24 h. After the reaction was complete, the DMF was removed by rotary evaporation. Distilled water was added to dissolve the product, which was then transferred to a dialysis bag for 2 days of dialysis. Finally, the product was freeze–dried to obtain the final product. PPDD was stored at 4°C away from light.

#### Characterization of PPDD

Agilent Cary 60 was used to determine the UV absorption spectrum. The absorption spectra of 200-800 nm were measured by UV spectrophotometer after weighing 10 mg of different products. A Fourier infrared spectrometer (Zhongke Ruijie (Tianjin) Technology Co., Ltd.) was used to measure the infrared spectrum of the material. ^1^H NMR spectra were recorded on a Bruker AVANCE Neo 400WB spectrometer using deuterated dimethyl sulfoxide (DMSO-d_6_) as solvent at room temperature. Chemical shifts (δ) are reported in parts per million (ppm), and coupling constants (J) are given in Hertz (Hz).

#### Cell experiments

Each 96-well plate was added with 100 µL of 5000 cells and cultured until the cells were fully attached to the wall. Then 10 µL of different drugs were added to stimulate the cells, followed by 10 µL of the CCK-8 solution in each well. The cells were incubated for 0.5 h, and the absorbance at 450 nm was measured using an enzyme-labeler (Thermo Fisher Technology Co., LTD.). For the confocal cell culture dish, 10^5^ cells were added to 1 mL of medium and cultured until the cells were fully attached to the wall. After administering 100 µL of 100 µg/mL drug to stimulate the cells, the cells were incubated for 1-4 h. LSM980 (Carl Zeiss AG, Germany) was used for imaging under the 488 nm laser channel. DCFH-DA was diluted in serum-free medium at 1:1000 to a final concentration of 10 μmol/L. The cell culture medium was removed and 20 µL of diluted DCFH-DA was added. Subsequently, the cells were incubated at 37°C for 20 min. The cells were washed three times with serum-free cell culture solution to completely remove DCFH-DA that did not enter the cells. LSM980 was used for imaging 405- and 488-nm laser channels. The cells were inoculated in 96-well plates. After the cells were attached to the wall, the culture medium was removed, and the cells were cleaned twice with PBS. Then, 100 μL of Calcein AM staining solution was added, and the dye was gently shaken to evenly cover all cells. After incubation, the cells were replaced with fresh preheated culture medium at 37°C, incubated at 37°C for another 30 min in the darkness, removed the culture medium, cleaned with PBS 3 times, added serum-free cell culture medium, and observed under a fluorescence microscope. For in vitro anti-inflammatory assessment, macrophages were seeded onto confocal dishes and stimulated with LPS and various treatments as indicated. For NO detection, cells were incubated with 5 μmol/L DAF-FM DA at 37°C for 20 min, washed 3 times with serum-free medium, and imaged using a confocal microscope under 488 nm excitation. Nitrite levels in supernatants were quantified using a Griess reagent assay kit (Beyotime Biotechnology). Levels of TNF-α, IL-6, and IL-10 were determined using commercial ELISA kits according to the manufacturer’s instructions.

#### In vivo experiments

The mouse colon was exposed and punctured with a syringe. The abdominal cavity was then sutured, and LPS was injected into the abdomen. Cy5-labelled PPDD was administered to septic mice, which were anaesthetized with isoflurane and imaged at Pre, 30, 60, 120 and 240 min with an IVIS Lumina III system. At 1, 2, 4, 8, 12, 24, 48 and 96 h, mice were euthanized, the heart, liver, spleen, lungs and kidneys were excised, rinsed in PBS and imaged ex vivo under the same IVIS parameters. Blood was collected by cardiac puncture, serum was separated by centrifugation and concentrations of TNF-α, IL-6, IL-10 and CRP were determined in duplicate using commercial ELISA kits following the manufacturers’ protocols; absorbance was read at 450 nm and values obtained from standard curves. Mouse heart tissue samples were removed, and frozen slices were made with a thickness of 10 µm each. After ROS staining, a small amount of DAPI staining solution was added to the sample surface, and the sample was incubated at room temperature for 5 min. The DAPI staining solution was removed, and the samples were washed with PBS 3 times, for 5 min each. At the end of the treatment period, the mice were dissected and their major organs (heart, liver, spleen, lungs, and kidneys) were fixed in 10% formalin. The organs were then sliced and immunohistochemically stained with H&E. To further evaluate the biocompatibility of the PPDD, we adopted the semiquantitative histopathology scores [45]. The scoring criteria were as detailed in Supplementary Tables S1.

## Results and discussion

### Synthesis of PPDD

**Figure 2.**
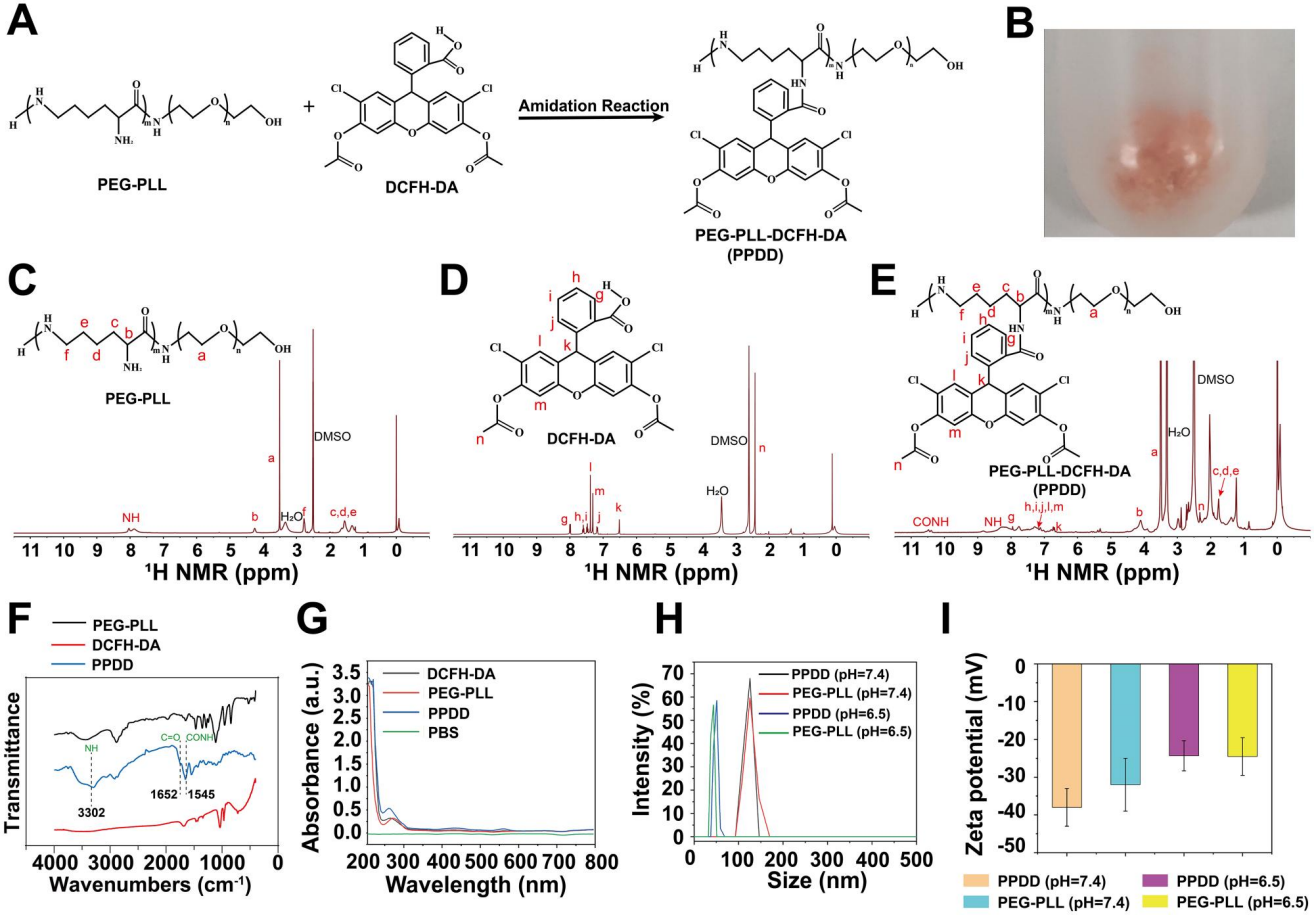
Material characterization of PPDD. (**A**) PEG-PLL and DCFH-DA synthesize PPDD by amide bonding. (**B**) PPDD physical digital photos. (**C**) ^1^H NMR of PEG-PLL. (**D**) ^1^H NMR of DCFH-DA. (**E**) ^1^H NMR of PPDD. (**F**) FT-IR spectroscopy of PEG-PLL, DCFH-DA and PPDD. (**G**) UV absorption spectra of PEG-PLL, DCFH-DA and PPDD. (**H**) Size of PEG-PLL and PPDD. (**I**) Zeta potential of PEG-PLL and PPDD. Data were expressed as mean ± standard deviation (SD) (*n* = 3).

PEG-PLL reacts with DCFH-DA through amide bonding to form PPDD (Figure 2A). PPDD is orange–yellow at room temperature (Figure 2B). H nuclear magnetic resonance spectroscopy (^1^H NMR) analyses of PEG-PLL, DCFH-DA and PPDD (Figure 2C–E) confirmed the copolymer structure and the amidation reaction for PPDD synthesis was successfully implemented. Specifically, the ^1^H NMR spectrum of PPDD displays a new singlet at δ 10.5 ppm derived from the amide CONH proton, while simultaneously retaining all characteristic resonances of PEG-PLL and DCFH-DA, altogether providing unambiguous evidence that the conjugation proceeded to completion and that PPDD was successfully synthesized (detailed ^1^H NMR data are provided in Supplementary Tables S2–S4). The Fourier transform infrared (FTIR) spectroscopy results indicated the presence of amide bonds in PPDD, further confirming the successful chemical conjugation between PEG-PLL and DCFH-DA (Figure 2F). Analysis of ultraviolet (UV) absorption spectra from different experimental groups revealed that PPDD exhibits a distinct peak at 260 nm, coinciding with the characteristic peak of PLL at the same wavelength (Figure 2G). These experiments provide compelling evidence for the successful synthesis of PPDD.

### Acidity-activated structural transformation

To verify the self-assembly properties of PEG-PLL and PPDD copolymers, their particle size and zeta potential at various pH levels were monitored, exploring acidity-activated structure transformation. It is observed that DCFH-DA conjugation had neglectable influence on PEG-PLL self-assembly, as both PEG-PLL and PPDD exhibited an approximately identical hydrated particle size (∼100 nm) at pH 7.4 (Figure 2H), thereby validating the spontaneous self-assembly ability of PEG-PLL and PPDD. Notably, the particle sizes of PPDD and PEG-PLL in buffer at pH 6.5 were significantly smaller than those measured in buffer at pH 7.4, indicating the successful acidity-activated structural transformation of PEG-PLL and PPDD copolymer assemblies via disassembly and reconfiguration. Mechanistically, under mildly acidic conditions, the pH-responsive sites within the copolymer react in distinct ways: ε-amino groups become protonated, whereas ester bonds are prone to acid-catalyzed hydrolysis. This acidity-activated structural transformation hydrophobic segments diminish intermolecular repulsion and intensify hydrophobic interactions, prompting partial disassembly of the initial assemblies and their reorganization into smaller, more compact nanostructures [46]. Accordingly, the acidity-activated structural transformation of PEG-PLL and PPDD assemblies resulted in alterations of their potential, as evidenced by a decrease in potential of PPDD and PEG-PLL in buffer at pH 6.5 compared with at pH 7.4 (Figure 2I).

**Figure 3.**
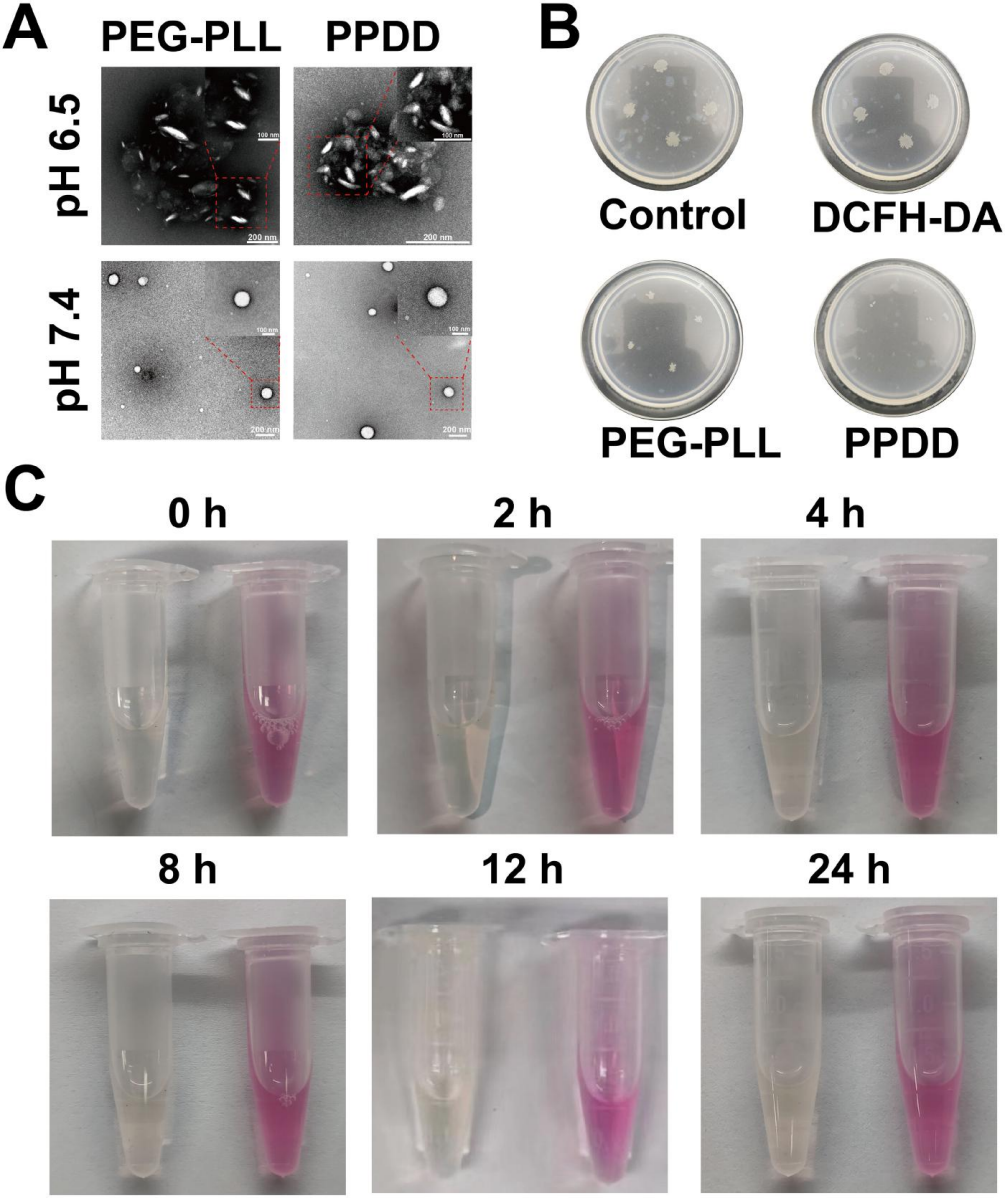
Bacteriostatic effect of PPDD. (**A**) TEM images of PEG-PLL and PPDD. (**B**) Bacteriostatic effects of PEG-PLL and PPDD. (**C**) Images of PPDD distribution over a long period in PBS and DMEM media.

Transmission electron microscopy (TEM) images revealed that, in high-pH buffer, the negatively charged PPDD and PEG-PLL assemblies electrostatically repelled each other, resulting in a highly dispersed population of spherical self-assemblies. Intriguingly, upon exposure to an acidic microenvironment, the acidity-triggered disassembly and reconfiguration rendered the spherical PEG-PLL and PPDD self-assemblies ellipsoidal, and the actual particle size of PEG-PLL and PPDD was approximately 50 nm (Figure 3A). This acidity-activated structural transformation exposes additional ROS-scavenging active sites and is, therefore, expected to enhance antibacterial activity, mitigate inflammation and treat sepsis, given the acidic sepsis microenvironment.

### *In vitro* antibacterial and oxidative stress removal tests

PLL possesses exceptional natural antibacterial properties. However, the extensive cellular uptake of PLL limits its further biological application because of its cytotoxicity. In cultures of *Escherichia coli*, PPDD also exhibited antibacterial properties, suggesting that it can effectively impede bacterial growth (Figure 3B). PPDD was dispersed in phosphate-buffered saline (PBS) (left) and Dulbecco’s modified Eagle medium (DMEM) (right). After a long-term observation, no precipitation was observed, indicating the long-term stability of PPDD in the liquid environment (Figure 3C). These results demonstrate that PPDD was successfully synthesized, retained the antimicrobial properties of PLL and exhibits long-term stability.

**Figure 4.**
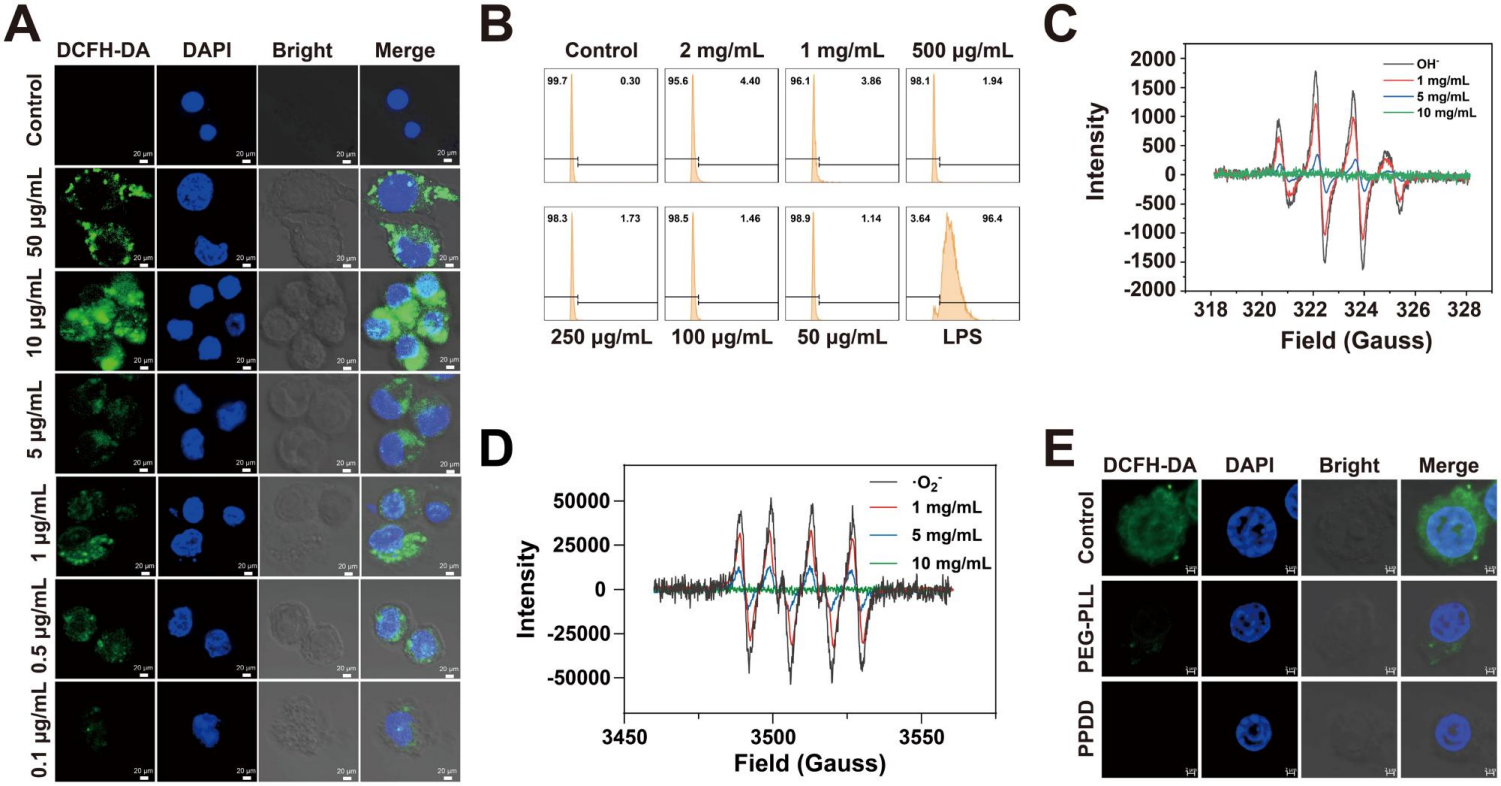
ROS removal properties of materials *in vitro*. (**A**) LSCM detection of ROS production in macrophages by LPS at different concentrations. (**B**) Flow cytometry of macrophages producing ROS with different concentrations of PPDD. ESR detection of OH^-^ (**C**) and ·$O_{2}^{-}$ (**D**) clearance with different concentrations of PPDD. (**E**) LSCM detection of ROS clearance in the control, PEG-PLL and PPDD groups.

Cells were exposed to varying concentrations of lipopolysaccharide (LPS) to mimic sepsis-associated cellular damage [14, 47], and a direct correlation between LPS concentration and intracellular ROS levels was observed (Figure 4A). With increasing LPS concentrations, intracellular ROS levels rose gradually. At the highest concentration (50 μg/mL), LPS induced morphological changes in macrophages, indicating a potent stimulatory effect on these cells. Subsequently, the impact of PPDD on macrophages was evaluated. Flow-cytometry analysis revealed that macrophages produced minimal ROS at all tested PPDD concentrations, indicating a favorable safety profile for PPDD toward macrophages (Figure 4B). Electron spin resonance (ESR) analysis confirmed the remarkable ability of PPDD to scavenge ROS, as the PPDD concentration increased, the characteristic peak intensities of OH^−^ and ·$O_{2}^{-}$ diminished in a dose-dependent manner, demonstrating its outstanding efficacy in reducing oxidative stress (Figure 4C and D). Laser-scanning confocal microscopy (LSCM) experiments revealed that both PEG-PLL and PPDD effectively eliminate ROS. Notably, PPDD demonstrated superior ROS clearance because the conjugated DCFH-DA facilitates cell-membrane penetration, resulting in greater intracellular uptake, and thus, more efficient ROS scavenging (Figure 4E). Interestingly, PPDD yielded the weakest intracellular fluorescence at pH 6.5 (Supplementary Figure S1). The additional attenuation at pH 6.5 compared with pH 7.4 indicates acidity-activated PPDD reconfiguration and greater intracellular availability of scavenging sites, resulting in more efficient ROS scavenging.

### *In vitro* ROS scavenging evaluation

**Figure 5.**
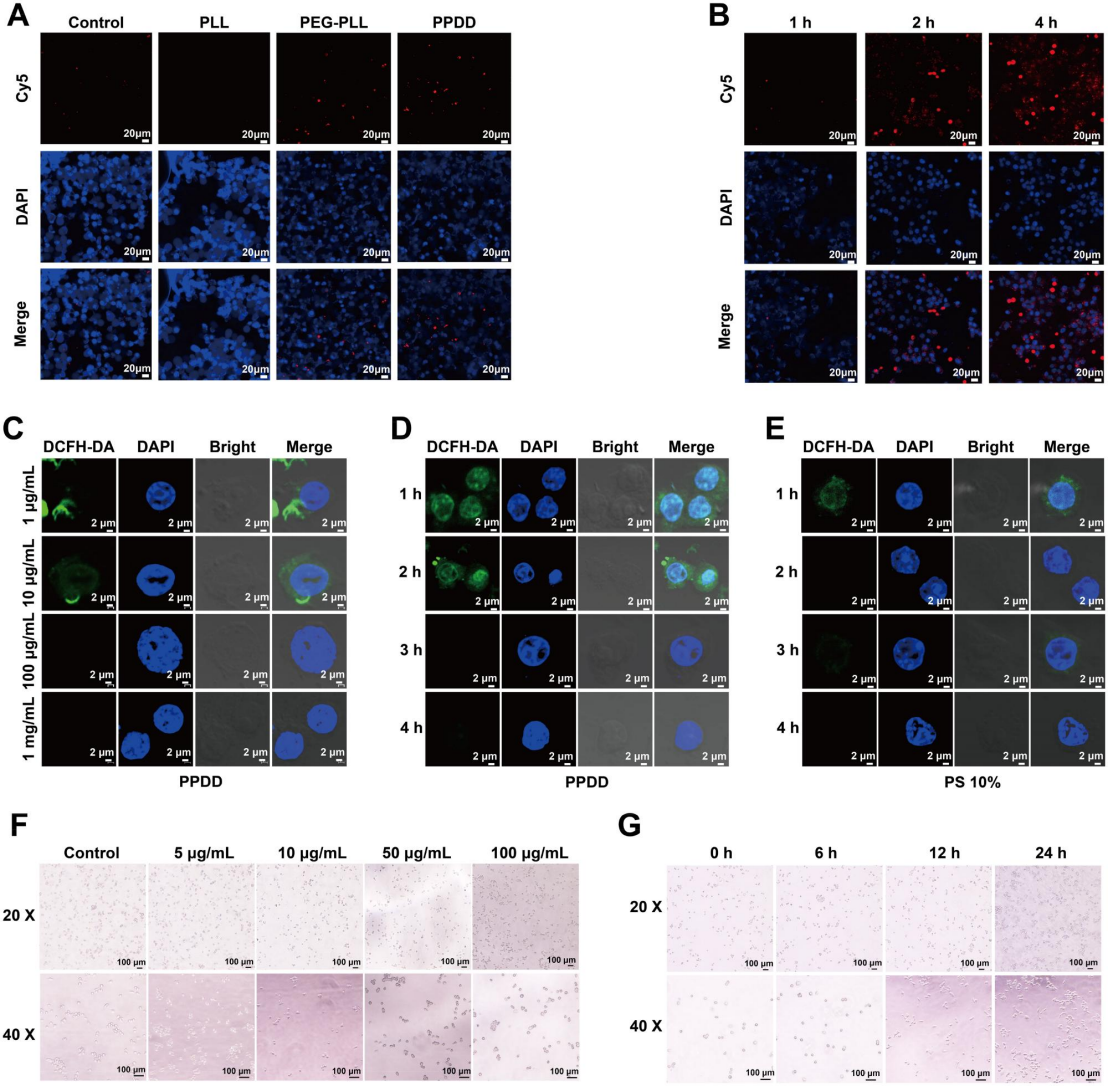
ROS Scavenging *in vitro*. (**A**) LSCM images of phagocytic cells after coincubation with control, PLL, PEG-PLL and PPDD. (**B**) Time-dependent LSCM images of cells after incubation with PPDD in different time-treated groups. (**C**) LSCM detection of ROS clearance at different concentrations of PPDD. (**D**) LSCM detection of clearing ROS by PPDD at different times. (**E**) LSCM detection of clearing ROS by PS 10% at different times. (**F**) Optical photographs of PPDD incubated with macrophages at different concentrations. (**G**) Optical photographs of PPDD incubated with macrophages at different times.

To investigate the cellular affinity for PPDD, we assessed PPDD phagocytosis across different treatment groups. The LSCM results revealed minimal intracellular fluorescence intensity of PLL because of its cytotoxicity. In contrast, the PPDD group exhibited more red fluorescence within cells than the PEG-PLL group, which was attributed to the stronger affinity effect of DCFH-DA on the cell membrane (Figure 5A). Moreover, LSCM showed stronger intracellular fluorescence for PPDD at pH 6.5 than at pH 7.4, indicating greater macrophage uptake (Supplementary Figure S2). This accords with reports that mildly anisotropic, slightly smaller oblate/ellipsoidal nanoparticles engage macrophage membranes with favorable orientation and larger contact edges, thereby promoting faster phagocytic internalization than larger spherical counterparts [48, 49]. Phagocytosis of PPDD was observed in cells, and the uptaken PPDD nanoparticles persistently retained over different periods (Figure 5B). To evaluate the ROS-scavenging capability of PPDD at different concentrations, LSCM observations were conducted at 1 mg/mL, 100 μg/mL, 10 μg/mL and 1 μg/mL, respectively. Imaging analysis revealed a gradual improvement in ROS clearance as the concentration level increased (Figure 5C). This result indicates that PPDD possesses a strong ability to eliminate ROS. To compare the cytoprotective effects of PPDD and 10% penicillin-streptomycin (PS) mixture on cells, ROS clearance images were taken over 1–4 h. The experimental findings suggest that the cytoprotective efficacy of PPDD is less effective than 10% PS at the initial stage (Figure 5D and E). However, long-term observations revealed that PPDD exhibits remarkable ROS clearance capabilities comparable to 10% PS. Under different PPDD concentrations and long-term incubations with macrophages, microscopic images revealed that the macrophages did not undergo any morphological changes. This result indicates that PPDD is highly biocompatible and does not exert strong biostimulatory effects on macrophages (Figure 5F and G).

### *In vitro* anti-inflammation test

**Figure 6.**
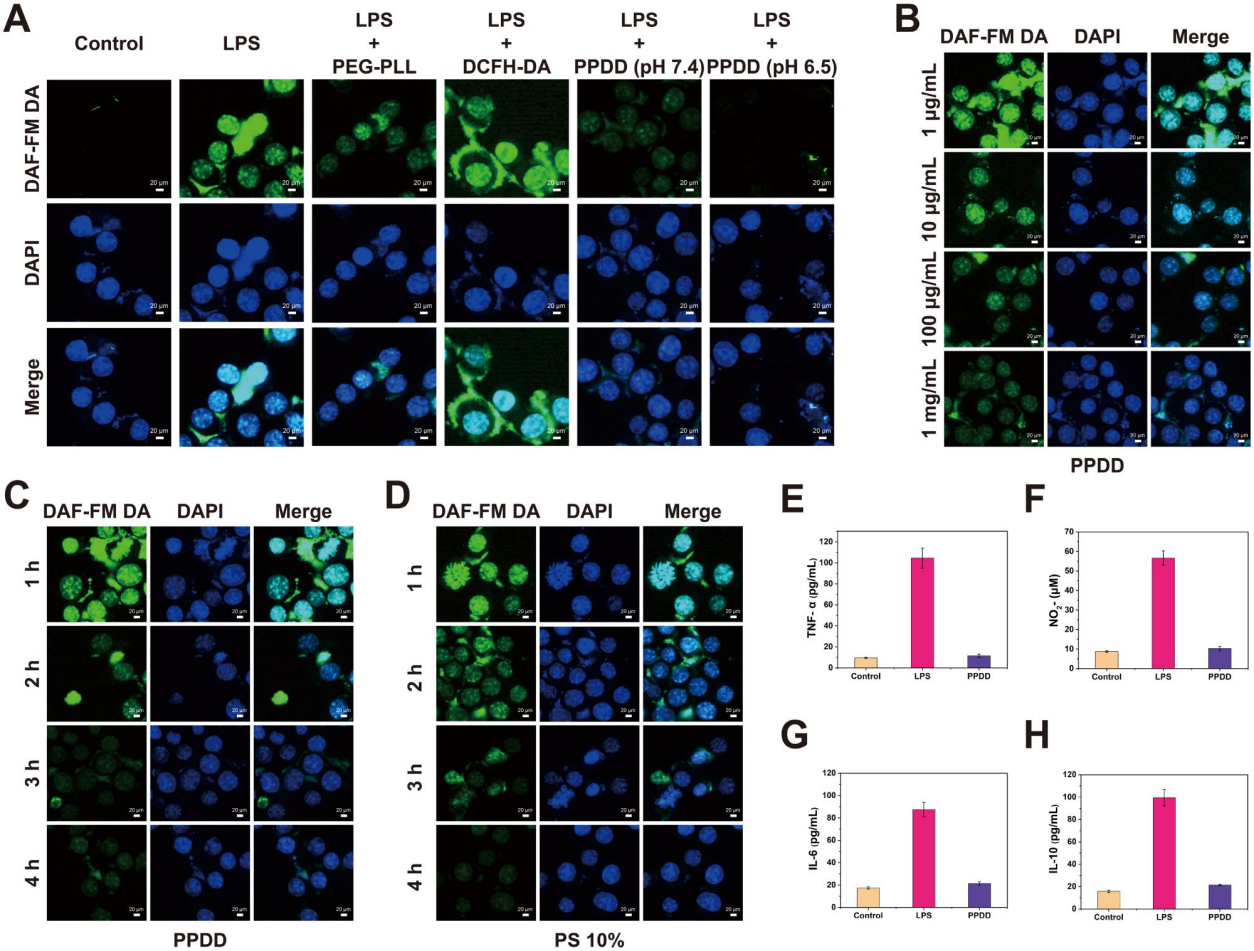
*In vitro* anti-inflammatory effects of PPDD on LPS-stimulated macrophages. (**A**) LSCM images of DAF-FM DA-labeled macrophages after different treatments. (**B**) Dose-dependent suppression of intracellular NO in macrophages treated with PPDD. (**C**) Time-dependent reduction of NO in macrophages incubated with PPDD for 1, 2, 3 and 4 h. (**D**) Time-dependent NO reduction in macrophages treated with 10% PS over the same interval. (**E**) TNF-α, (**F**) ${\text{NO}}_{2}^{-}$, (**G**) IL-6 and (**H**) IL-10 concentrations in macrophage supernatants after the indicated treatments (*n* = 3).

To assess the anti-inflammatory efficacy of PPDD *in vitro*, we examined its impact on NO production in LPS-stimulated macrophages. LPS provoked intense DAF-FM DA fluorescence, whereas PPDD sharply diminished the signal under both pH 7.4 and pH 6.5, the latter yielding the greatest suppression (Figure 6A). Increasing PPDD from 1 µg/mL to 1 mg/mL produced a graded reduction in intracellular NO, confirming a dose-dependent effect (Figure 6B). Time-lapse imaging revealed a steady decline in intracellular NO between 1 and 4 h, indicating sustained suppressive activity (Figure 6C). By contrast, a 10% PS control achieved comparable but not superior NO suppression within the same period (Figure 6D). Subsequent quantitative assessment corroborated these results, demonstrating that PPDD suppressed tumor necrosis factor-α (TNF-α), nitrite (${\text{NO}}_{2}^{-}$) and interleukin 6 (IL-6) to near-basal concentrations while restoring interleukin 10 (IL-10) toward homeostatic levels (Figure 6E–H).

### *In vitro* biosafety evaluation

The verification of PPDD's biosafety is a prerequisite for conducting animal experiments in academic research. Calcein acetoxymethyl ester (Calcein AM) was used to evaluate the cytotoxic effects of different experimental materials on cellular viability (Supplementary Figure S3A). The semi-quantitative results demonstrated that the synthesized PPDD maintained roughly 95% cell viability and did not elicit any discernible biological toxicity, confirming the safety of PPDD (Supplementary Figure S3B). Long-term incubation of cells with PPDD preserved 96% cell viability, further confirmed the safety of PPDD (Supplementary Figure S3C and D). At higher concentrations (10 mg/mL), PPDD still maintained 96% cell viability, causing no side effects on cell viability (Supplementary Figure S3E and F). Further biosafety testing of PPDD was performed using Cell Counting Kit-8 (CCK-8) experiments. Consistent results showed that even after prolonged incubation (12 h) or at higher dose (10 mg/mL), PPDD maintained at least 85% cell viability in HUVEC and macrophage cultures (Supplementary Figures S4 and S5).

### *In vivo* sepsis treatment and monitoring

**Figure 7.**
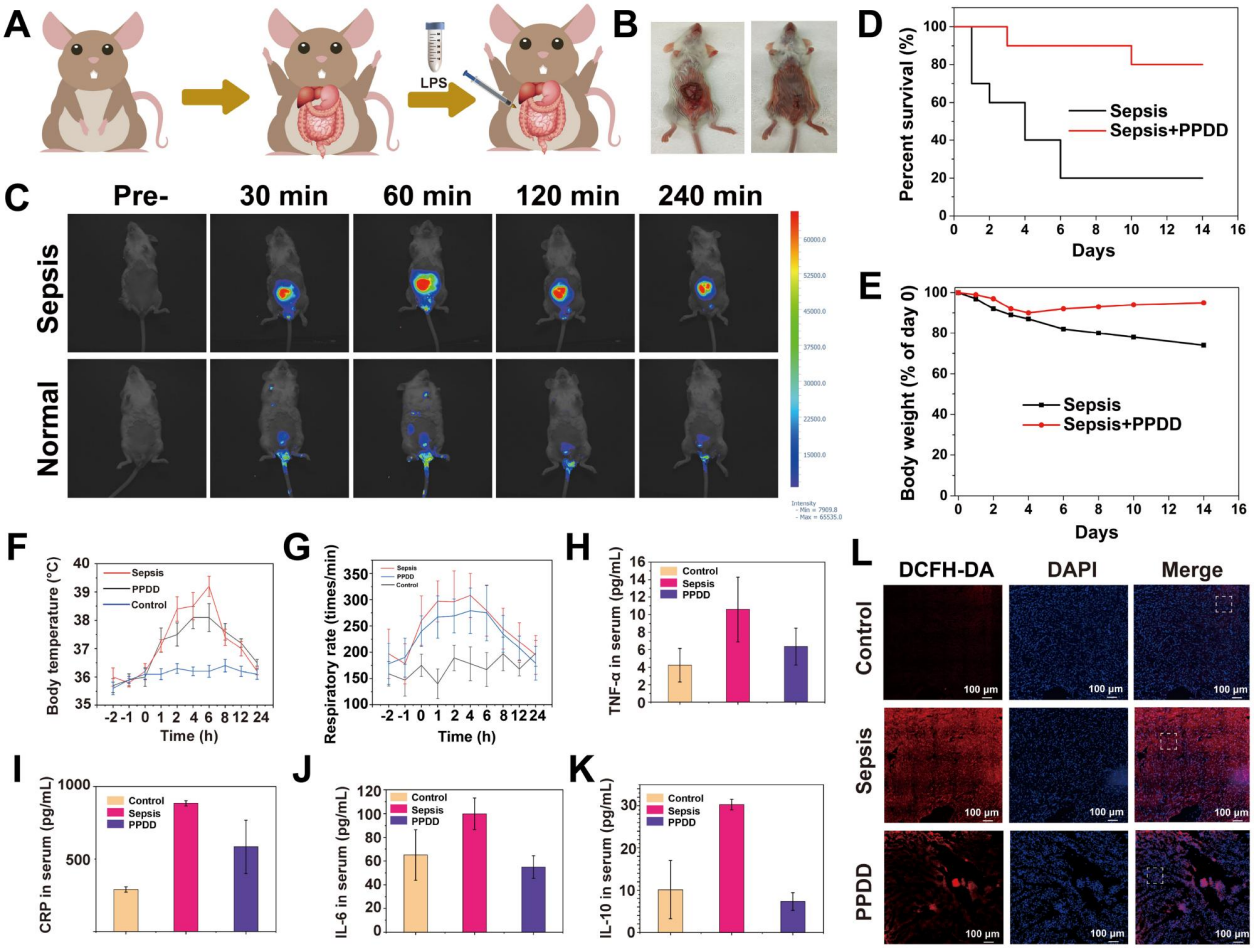
*In vivo* material verification of the accelerated healing in septic mice. (**A**) Schematic diagram of a septic mouse model. (**B**) Photos of mice with sepsis before and after modeling. (**C**) Time-dependent *in vivo* fluorescence images of septic and healthy mice that were intravenously injected with PPDD. (**D**) Survival rates and (**E**) body weight of septic mice that were treated with and without PPDD (*n* = 10). (**F**) Time-correlated temperature changes of mice with sepsis after different treatments. (**G**) Time-correlated respiratory rate changes of mice with sepsis after different treatments. Levels of TNF-α (**H**), CRP (**I**), IL-6 (**J**) and IL-10 (**K**) in serum of septic mice after different treatments. (**L**) LSCM images of septic tissues for detecting DCFH-DA signal in different treatment groups. Data were expressed as mean ± SD (*n* = 6).

All animal experiments were carried on under the approval of Animal Welfare Ethics Committee of Shanghai Tenth People’s Hospital (NO. SHDSYY-2023-Y3126-1). To further verify the *in vivo* biosafety of PPDD, healthy mice received injections of PBS or PPDD every other day for 14 days, and hematological and biochemical parameters measured on day 14 remained within physiological limits (Supplementary Figure S6). A mouse model of sepsis was developed by injecting colonic bacteria into the abdominal cavity using acupuncture to mimic sepsis onset (Figure 7A) [50, 51], and its successful establishment is shown in Figure 7B. Before pathological evaluation, *in vivo* fluorescence imaging was performed to monitor variations in oxidative stress and the progression of sepsis evolution after treatment (Figure 7C). No fluorescence signal was observed in healthy mice injected with PPDD. By contrast, the DCFH-DA fluorescence signal at the sepsis site in septic mice emerged and reached a maximum at 60 min, indicating that progressive accumulation of PPDD in the infected tissue. After 60 min, the DCFH-DA signal gradually declined, indicating that the degree of ROS scavenging in the PPDD group was significant. Subsequently, the gradual decline in signal indicates that PPDD is being cleared from the infection site and that local ROS levels are decreasing as the treatment takes effect, reflecting the dynamic accumulation kinetics and therapeutic action of PPDD in septic tissues. These results demonstrate that acidity-activated PPDD assembly can trace oxidative stress and monitor sepsis evolution. *Ex vivo* fluorescence imaging showed pronounced hepatic fluorescence within 1–12 h post-injection, indicating rapid liver accumulation of PPDD. The signal then declined progressively and was nearly undetectable by 96 h, demonstrating efficient systemic clearance, minimal risk of long-term tissue retention and supporting PPDD’s feasibility as a safe drug-delivery system (Supplementary Figure S7).

After monitoring the body weight and survival of the mice, PPDD treatment significantly prolonged the survival rate and recovered, the body weight of septic mice compared with the septic mice without treatment in the control group (Figure 7D and E). The statistical analysis showed a significant increase in body temperature and respiratory rate at 1 h after modeling, indicating the successful induction of sepsis in mice. Following the administration of PPDD via the tail vein, the temperature and respiratory rate of mice were monitored at various time points. The results demonstrated a more rapid recovery to normal levels post-treatment, indicating that PPDD exerts a powerful therapeutic effect in septic mice (Figure 7F and G). Enzyme-linked immunosorbent assay (ELISA) for TNF-α, C-reactive protein (CRP), IL-6 and IL-10 in the blood demonstrated that PPDD has a significant therapeutic effect (Figure 7H–K), and it normalizes the high inflammation cytokines in septic mice. The presence of leukocytes in the body is a crucial recovery indicator of immune response [52, 53]. Therefore, the quantity of leukocytes present in the bloodstream was assessed to yield experimental data demonstrating PPDD’s ability to expedite recovery. As expected, the results aligned with previous findings, affirming PPDD's efficacy in mitigating inflammation (Supplementary Figure S8). Furthermore, septic tissues were sliced and imaged and DCFH-DA conjugated in PPDD successfully detected ROS, demonstrating a decrease in oxidative stress after PPDD treatment (Figure 7L), confirming PPDD’s protective effect on the myocardium of mice with sepsis. To determine its accuracy, another ROS indicator, dihydroethidium (DHE), was used to stain the septic tissues, and identical results were obtained (Supplementary Figure S9), in which PPDD treatment reduces oxidative stress. H&E staining of organs indicated no toxic side effects from PPDD. No apoptosis, necrosis and cell density decline were observed (Supplementary Figure S10A–C). To quantitatively substantiate the histological observations, three board-certified pathologists independently performed blinded histopathology scoring. The assessment followed the grading guideline for tissue responses described by Mann PC et al (Supplementary Table S4). Semiquantitative histopathology scores for all organs were 0, further confirming the excellent biocompatibility of PPDD (Supplementary Figure S10D).

Nonetheless, before clinical use, the PPDD system must overcome several general limitations of polymeric nanoparticles. In particular, its *in vivo* pharmacokinetics and biodistribution will need optimization, since even minor formulation changes can dramatically alter tissue distribution and tolerability. Large-scale manufacturing under Good Manufacturing Practice (GMP) conditions will also be challenging: producing uniform PPDD at industrial scale requires tight control of particle size, charge and composition to ensure batch-to-batch consistency. Finally, comprehensive studies of long-term safety and efficacy in advanced animal models will be essential, with full ADME (absorption-distribution-metabolism-excretion) profiling and toxicological evaluation to support translation into human trials [54].

## Conclusion

In summary, we successfully engineered an acidity-activated PPDD by conjugating PEG-PLL with DCFH-DA. With the bactericidal and antioxidant properties of PLL, PPDD successfully targeted the pathogenesis of sepsis to remove oxidative stress, mitigate inflammation and kill infected bacteria. More significantly, the disassembly and reconfiguration of the spherical PPDD copolymer self-assembly are initiated by the acidic sepsis microenvironment and are reassembled into an ellipsoidal structure. This acidity-activated structural transformation exposes additional bioactive sites capable of scavenging ROS, further enhancing oxidative stress removal, bactericidal activity, inflammation mitigation and sepsis alleviation. Owing to these effects, bacterial reduction, oxidative stress attenuation and inflammation resolution were validated, which together led to sepsis alleviation and improved survival. Additionally, the conjugated DCFH-DA enables PPDD to monitor post-treatment variations in oxidative stress and sepsis, permitting prognosis evaluation. Briefly, the pharmaceutical PPDD offers the distinct advantages of high biosafety and an integrated diagnostic–therapeutic approach.

## Experimental section

All experimental details, methods, materials and Supplementary Figures S1–S10 are included in Supporting Information.

## Supplementary Material

rbaf100_Supplementary_Data
